# Light-activated viologen-based metal-covalent organic framework sprayable hydrogel for accelerated healing of infected skin wounds

**DOI:** 10.3389/fcimb.2026.1975470

**Published:** 2026-10-14

**Authors:** Li-Jun Feng, Jia-Ling Tong, Qin-Xie Xie, Mao-Nan Wen, Xiao-Zhao Ma, Yue Zang, Jia-Ru Zhong, Jia Yao, Xiao-Ming Li, Bo Zhao, Pei Tang

**Affiliations:** 1 Laboratory of Tumor and Immunity, School of Basic Medical Sciences, Guangdong Pharmaceutical University, Guangzhou, China; 2 The First Affiliated Hospital, Jinan University, Guangzhou, China; 3 Dongguan Key Laboratory of Traditional Chinese Medicine Hepatology, the Affiliated Dongguan Songshan Lake Central Hospital, Guangdong Medical University, Donggguan, China; 4 School of Pharmaceutical Sciences, Guangzhou Medical University, Guangzhou, Guangdong, China

**Keywords:** metal-covalent organic frameworks, viologen, antibacterial photodynamic therapy, sprayable hydrogel, infected wounds

## Abstract

Bacterial resistance and recurrent infection remain major challenges for infected wound healing. Antibacterial photodynamic therapy (aPDT) features excellent spatiotemporal control and low risk of inducing bacterial resistance, making it a promising antibacterial modality. However, many photosensitizers used in wound therapy are photounstable, bind weakly to bacteria, and generate insufficient reactive oxygen species (ROS). We developed a viologen-functionalized metal-covalent organic framework (Vio-MCOF) by covalently embedding viologen units into the MCOF framework, and integrated this material into a sprayable, ionically crosslinked sodium alginate hydrogel. In this system, viologen units serve as electron mediators and work cooperatively with the copper-cluster active sites to suppress charge recombination and may increase ROS production under simulated solar light. The positive charge of the viologen units promotes electrostatic binding of Vio-MCOF to negatively charged bacterial membranes, improving its photodynamic antibacterial performance. *In vitro* experiments showed that Vio-MCOF at 200 μg mL^-1^ achieved a bactericidal rate of over 99% against *S. aureus* after 30 min of simulated solar light irradiation. *In vivo* experiments demonstrated that the Vio-MCOF hydrogel reduced bacterial colonization in infected wounds, suppressed TNF-α and IL-6 expression, alleviated inflammation, and promoted angiogenesis and collagen deposition, leading to accelerated wound healing. Collectively, the sprayable Vio-MCOF hydrogel holds promise as an antibiotic-free dressing for infected skin wounds.

## 1 Introduction

When the skin barrier is compromised, wounds become susceptible to bacterial colonization. In infected wounds, bacteria can sustain a pro-inflammatory microenvironment and interfere with tissue remodeling, which often converts acute wounds into chronic lesions (Almadani et al., 2021; Peña and Martin, 2024). Studies have demonstrated that more than 90% of chronic wounds are accompanied by bacterial infection. Without effective treatment, bacteria can form persistent biofilms and cause severe complications including bacteremia and septic shock (Li et al., 2021; Uberoi et al., 2024). Conventional antibiotic therapies and wound dressings show limited efficacy against drug-resistant bacteria and biofilm-associated infections. Long-term antibiotic use can accelerate bacterial resistance, leading to wound recurrence, prolonged inflammation, and impaired healing (Uberoi et al., 2024; Guo et al., 2022b). Thus, developing effective non-antibiotic strategies is essential for managing infected wounds (Kibungu et al., 2021; Nasra et al., 2023).

Antibacterial photodynamic therapy (aPDT) has received increasing attention for infected wound management because light irradiation allows spatiotemporal control of antibacterial action and may reduce the selection pressure associated with antibiotic resistance (Dong et al., 2025; Zhang et al., 2023; Li et al., 2019). Under simulated solar light irradiation, photosensitizers produce reactive oxygen species (ROS), which trigger bacterial oxidative stress by damaging membranes, proteins, and genetic material to cause bacterial death (He et al., 2024; Xie et al., 2023). However, conventional small-molecule photosensitizers suffer from limitations, including poor photostability, rapid photogenerated charge recombination, low ROS production yield and weak bacterial adhesion, which restrict their use in complex infected wounds (Liu et al., 2023b; Chen et al., 2022). Therefore, photosensitizers with efficient light utilization, good stability, and strong bacterial binding are needed to improve aPDT (Zhao et al., 2022; Ren et al., 2026).

Viologen is a cationic redox-active unit with visible-light absorption and reversible electron-transfer properties (Zhou et al., 2025b). It can function as an electron mediator to accelerate photogenerated charge separation, while its permanent positive charge generates electrostatic attraction to negatively charged bacterial membranes. This effect reduces ROS diffusion distance and improves bactericidal specificity (Guo et al., 2022a). However, free viologen small molecules show high water solubility and poor structural stability. These molecules readily diffuse away from the wound site and leach into surrounding tissues. Such leakage raises biosafety concerns and limits their use in wound repair. Covalently anchoring viologen moieties into crystalline porous frameworks provides a promising strategy to overcome the limitations of free viologen molecules while preserving their photophysical and bacterial-binding properties (Das et al., 2017).

Metal-organic frameworks (MOFs) and covalent organic frameworks (COFs) are two major categories of crystalline porous materials widely explored in biomedicine and photocatalysis (Wang et al., 2024). MOFs possess high surface areas and abundant metal active sites but suffer from weak coordination bonds that are prone to structural collapse under physiological conditions (Ren et al., 2023; Zhu et al., 2022). In contrast, COFs are constructed via robust covalent linkages with structural stability, but the absence of efficient metal catalytic centers leads to rapid photogenerated electron-hole recombination and limited photodynamic activity. To address these limitations, metal-covalent organic frameworks (MCOFs) have been developed to integrate the complementary advantages of MOFs and COFs. MCOFs possess stable covalent frameworks and accessible Cu-cluster active sites, which facilitate efficient separation of photogenerated charge carriers and tunable photoresponse while maintaining good stability under physiological environments. These properties make MCOFs promising candidates for photodynamic antibacterial applications. However, viologen-functionalized MCOFs for solar-driven antibacterial wound therapy have rarely been reported.

In this study, we prepared Vio-MCOF by covalently incorporating cationic viologen units into the MCOF framework through aldehyde-amine condensation. The viologen units can capture and transfer photogenerated electrons, while the Cu-cluster sites participate in charge separation. Under simulated solar light, Vio-MCOF showed enhanced ROS generation. The positive charge of the viologen units promotes electrostatic binding to bacterial membranes, allowing Vio-MCOF to accumulate near bacteria and shorten the distance for ROS-mediated killing (Ren et al., 2026). We further prepared a sprayable, ionically cross-linked hydrogel by incorporating Vio-MCOF into a sodium alginate matrix. The hydrogel sets rapidly *in situ* and conforms to irregular wound surfaces (Zhou et al., 2025a; Lin et al., 2025). Compared with free photosensitizers, the composite system is more stable and captures bacteria more effectively (Liu et al., 2023c). This dressing provides photodynamic antibacterial, anti-inflammatory, and pro-angiogenic effects to promote infected wound repair (Fang et al., 2023; Zhou et al., 2025c).

## 2 Materials and methods

### 2.1 Materials

1,1’-Bis(4-aminophenyl)-[4,4’-bipyridine]-1,1’-dichloride (Vio-NH_2_), 4,4’’-diaminotetraphenyl (DAQP), and 1H-pyrazole-4-carbaldehyde were purchased from Shanghai Tensus Biotech Co., Ltd. Copper nitrate [Cu(NO_3_)_2_], calcium chloride (CaCl_2_), sodium sulfate (Na_2_SO_4_) and acetic acid (HAc) were purchased from Shanghai Macklin Biochemical Co., Ltd. Sodium alginate (SA) was purchased from Shanghai Aladdin Biochemical Technology Co., Ltd. Ketjen black was purchased from Guangdong Zhuguang New Energy Technology Co., Ltd. Phosphate buffer solution (PBS) and normal saline were purchased from Wuhan Servicebio Technology Co., Ltd. N,N-dimethylformamide (DMF) and ethanol (EtOH) were purchased from Tianjin Damao Chemical Reagent Factory. The Cell Counting Kit-8 (CCK-8) assay kit was purchased from Nanjing Xinshi Biotechnology Co., Ltd. the Dulbecco’s Modified Eagle Medium (DMEM) and fetal bovine serum (FBS) were both purchased from Gibco, Thermo Fisher Scientific. All chemical reagents were used as received without further purification.

### 2.2 Characterization

The morphology of the materials was observed by scanning electron microscopy (SEM, Gemini SEM 560, ZEISS). The crystal structure was characterized by X-ray diffraction (XRD, D8-Advance, Bruker) with Cu Kα radiation at 40 kV and 40 mA. Infrared spectra of the materials in the range of 4000–400 cm^-1^ were measured using Fourier Transform Infrared Spectroscopy (FT-IR, Nicolet iS20, Thermo Fisher). X-ray photoelectron spectroscopy (XPS, ESCALAB Xi+, Thermo Fisher) was used to analyze the elemental composition and chemical valence states of the materials. The solid UV-visible absorption spectrum was obtained by UV-Visible near-infrared spectrophotometer (UV-Vis-NIR, Lambda1050+, PerkinElmer). The detection wavelength range was 300–1500 nm, and the bandgap was explored. The Zeta potentials were characterized by Malvern Nano particle size potential analyzer (ZS90, Zetasizer Nano ZS). Electrochemical tests were performed using the CHI660E electrochemical workstation.

### 2.3 Synthesis of Cu_3-_cluster

30 mmol (2.88 g) 1H-pyrazole-4-carbaldehyde and 30 mmol (5.63 g) copper nitrate were mixed and dissolved in the mixture of 67 mL N, N-DMF, 50 mL H_2_O and 67 mL ethanol in a 250 mL pressure-resistant flask. The device was then placed in the oil bath and heated at 110 °C for 36 h. The obtained light yellow crystalline product was filtered, washed with deionized water three times, and vacuum-dried for further use.

### 2.4 Syntheses of DAQP-MCOF and Vio-MCOF

For the synthesis of DAQP-MCOF, a mixture solution of 4 mL DMF and 172 μL (3 M) glacial acetic acid was prepared, and 4,4’’-diaminotetraphenyl (DAQP, 0.15 mmol, 50.5 mg), and Cu_3_ (0.1 mmol, 47.6 mg) were added to it. The resulting mixture was thoroughly homogenized using a homogenizer, and any bubbles were removed by ultrasonic treatment prior to heating at 120 °C for 2 h. After the reaction, the mixture was washed twice with deionized water, DMF, and ethanol, respectively, and dried under vacuum to yield a dark-green powder.

For the synthesis of Vio-MCOF, a mixture solution of 6 mL DMF and 258 μL (3 M) glacial acetic acid was prepared, and 1,1’-bis(4-aminophenyl)- [4,4’-bipyridine]-1,1’-dichloride (Vio-NH_2_, 0.15 mmol, 62 mg), and Cu_3_ (0.15 mmol, 71.4 mg) were added to it. The remaining procedures were identical to those described above, finally yielding a blackish-brown powder.

### 2.5 Electrochemical measurements

The electrochemical test was performed in the standard three-electrode system of the CHI660E electrochemical workstation. Pt was used as the counter electrode, Ag/AgCl as the reference electrode, and Na_2_SO_4_ (0.1 M) as the electrolyte. 3 mg MCOF and 1 mg Ketjen black were dispersed into a mixture of 0.8 mL ethanol and 0.2 mL Nafion, and 60 μL of the above suspended droplets were added to the electrode surface (1×1 cm^2^) of the ITO glass base. The working electrode was prepared by drying at room temperature. Among them, the Mott-Schottky measurement was performed via the impedance-potential (IMPE) mode. The test frequency ranged from 1500 to 3000 Hz. Transient photocurrent responses under periodic visible-light on/off illumination were recorded using the amperometric i-t technique. Light-dark switching intervals were set to 10 s for measurements under simulated solar light and dark conditions.

### 2.6 Synthesis and characterization of MCOF-based spray hydrogels

By spraying solutions A and B onto each other, a sprayable gel dressing containing MCOF was formed. Solution A was a sodium alginate/PBS solution (SA, 1.5 wt%), containing 2 mg mL^–1^ of MCOF, while Solution B was a CaCl_2_ aqueous solution (3 wt%). When the two solutions meet at the wound site, the carboxyl groups in the sodium alginate undergo ionic crosslinking with calcium ions (Ca^2+^), rapidly forming a hydrogel network within approximately 5 s that encapsulates the material.

### 2.7 Evaluation of antibacterial activity of MCOF

The antimicrobial activity of the MCOF against *Staphylococcus aureus* (*S. aureus*) and *Ecvherichia coil* (*E. coil*) was tested using the colony-forming unit (CFU) method. *Escherichia coli* (ATCC 25922) and *S. aureus* (ATCC 25923) were both purchased from Microbiologics (USA). The bacteria were diluted with normal saline to a concentration of 1 × 10^7^ CFU mL^-1^. DAQP-MCOF and Vio-MCOF were added to 1 mL of bacterial solution to achieve final concentrations of 0, 25, 50, 100, and 200 µg mL^-1^. The experimental group was exposed to simulated solar light (a 300 W xenon lamp with an AM1.5 filter, 110 mW cm^-2^) for 30 min. In contrast, the control experiment was conducted in the dark for the same duration. Subsequently, 100 µL of the diluted bacterial suspension was inoculated onto LB agar plates. After incubation at 37 °C for 24 h, the colonies were counted, and the relative survival rate and antibacterial rate of the bacteria were determined based on CFU. All data represent the mean ± SD from three independent experiments.

### 2.8 Evaluation of antibacterial activity of hydrogels

The antibacterial efficacy of the hydrogels against *S. aureus* was tested using the CFU method. A 500 µL sample of the hydrogel was immersed in 75% ethanol for 1 h to sterilize it. After sterilization, the hydrogel was rinsed with normal saline to remove residual ethanol. Subsequently, the hydrogels were placed in a 24-well plate containing 500 µL of bacterial suspension (1 × 10^7^ CFU mL^-1^) and incubated for 30 min under simulated solar light conditions (300 W xenon lamp with an AM1.5 filter, 110 mW cm^-2^) and in the dark. Wells without hydrogel served as the control group. Subsequently, 100 µL of a diluted bacterial suspension was inoculated onto LB agar plates, which were incubated at 37 °C for 24 h. The number of colonies on the agar plates was counted, and the relative bacterial survival rate was determined based on CFU. All data represent the mean ± SD from three independent experiments.

### 2.9 Hemolysis assay

The hemocompatibility of Blank gel and MCOF gel was evaluated via an *in vitro* hemolysis assay. Fresh mouse orbital blood was collected in anticoagulant tubes and centrifuged at 3000 rpm for 10 min. The upper plasma layer and middle leukocyte-platelet layer were discarded, and the precipitated red blood cells (RBCs) were collected. The RBC precipitate was resuspended in 2 mL of normal saline and centrifuged at 3000 rpm for 10 min at 4 °C. The RBCs were repeatedly washed with normal saline until the supernatant became clear. A 2% RBC suspension was prepared by diluting the purified RBC with normal saline. Subsequently, 100 µL of the Blank gel and the MCOF gel samples were immersed in a mixture of 0.5 mL of RBC suspension and 0.5 mL of normal saline, respectively, to serve as the experimental group. Deionized water mixed with an equal volume of RBC suspension served as the positive control, while normal saline mixed with equal volume of RBC suspension was set as the negative control. All groups were incubated in a water bath at 37 °C for 2 h. After incubation, the mixtures were centrifuged at 3000 rpm for 10 min. Then, 100 μL of the supernatant from each sample was transferred into a 96-well plate, and the optical density at 542 nm was measured using a microplate reader. The hemolysis ratio was calculated using the following equation:


Hemolysis ratio (%)=(As−A0)/(Ab−A0)×100%

where As, Ab, and A0 correspond to the optical density of hydrogel sample groups, positive control groups, and negative control groups, respectively.

### 2.10 *In vitro* cytocompatibility evaluation

The *in vitro* cytocompatibility of Vio-MCOF powder was evaluated using mouse fibroblast L929 cells (Nasra et al., 2023). Cells were cultured in complete medium supplemented with 10% fetal bovine serum (FBS) and 1% penicillin-streptomycin, and maintained in an incubator at 37 °C, 5% CO_2_, and saturated humidity. L929 cells were seeded into 96-well plates at a density of 8×10^3^ cells/well, and cultured for 24 h to allow full adhesion. Subsequently, the original culture medium was removed and replaced with complete medium containing the powder at different concentrations, and cells were incubated for an additional 24 h. After incubation, CCK-8 working solution (10% of the medium volume) was added to every well. The plates were incubated at 37 °C in the dark for 2 h. The optical density (OD) values at 450 nm were measured using a microplate reader, and the cell viability was calculated according to the following formula.


Cell viability (%)=(As–Ab)/(Ac−Ab)×100%

where As, Ac and Ab represent the absorbance of sample wells, cell-only control wells, and blank medium wells, respectively.

### 2.11 *In vivo* animal wound-healing assays

To evaluate the therapeutic efficacy of the Vio-MCOF hydrogel in infected cutaneous wound healing, *in vivo* wound healing experiments were conducted using male C57BL/6 mice weighing 18–22 g. All animal procedures were approved by the Institutional Animal Care and Use Committee of South China Agricultural University (Approval No. 2026c068). Prior to the experiment, all mice were acclimatized under standard housing conditions and were randomly divided into three groups (n=5 per group): the control group (Control), the Vio-MCOF hydrogel group (Dark-Gel), and the Vio-MCOF hydrogel plus light irradiation group (Light-Gel).

Mice were anesthetized by intraperitoneal injection of sodium pentobarbital (30 mg/kg; 1% w/v working solution). After dorsal shaving and routine skin disinfection, a 10 mm full-thickness cutaneous wound was created on the dorsal skin using a sterile biopsy punch. Subsequently, 30 μL of a *S. aureus* suspension (10^6^ CFU mL^-1^) was topically inoculated onto each wound to establish an infected wound model (Liu et al., 2020). 24 h after bacterial inoculation, mice received the assigned treatments. Wounds in the Dark-Gel group were sprayed with Vio-MCOF-containing precursor solutions to form an *in situ* hydrogel, whereas wounds in the Light-Gel group received the same spraying treatment followed by light irradiation for 30 min. The Control group received no hydrogel treatment. Standardized digital photographs of the wounds were obtained on Days 0, 3, 6, 8, and 10. To assess the *in vivo* antibacterial efficacy, mice were deeply anesthetized and then euthanized by cervical dislocation on day 3 post-treatment, and wound tissues together with adjacent skin were aseptically harvested. The tissues were weighed, homogenized in sterile saline, and serially diluted. Aliquots of the tissue homogenates were spread onto LB agar plates and incubated at 37 °C for 18–24 h. The bacterial burden was quantified by colony counting and expressed as colony-forming units per gram of tissue (CFU/g tissue).

For histological evaluation, mice from another independent batch were similarly deeply anesthetized and then euthanized by cervical dislocation on days 3 and 10 post-treatment. Wound tissues were harvested and fixed in 4% paraformaldehyde for 12–24 h. After dehydration and paraffin embedding, the tissue samples were sectioned into continuous slices. Hematoxylin and eosin (H&E) staining, Masson’s trichrome staining, and immunohistochemical staining were subsequently performed. All stained sections were observed and photographed under an optical microscope.

### 2.12 Statistical analysis

Data analysis was performed using GraphPad Prism 8.0 software. All data were presented as mean ± standard deviation (SD). Comparisons between two groups were analyzed using Student’s t-tests, whereas one-way ANOVA was applied for multiple group comparisons. Statistical significance was defined as *p < 0.05, **p < 0.01, and ***p < 0.001.

## 3 Results

### 3.1 Synthesis and characterization of MCOF

Two novel metal-covalent organic frameworks (MCOFs), designated as DAQP-MCOF and Vio-MCOF, were successfully synthesized via a solvothermal method using Cu_3_ as the metal node, with DAQP or Vio-NH_2_ serving as organic linkers through condensation reactions (**Figure 1A**). The crystal structures of the obtained materials were first analyzed by powder X-ray diffraction (PXRD). As shown in **Figure 1B**, DAQP-MCOF exhibited distinct diffraction peaks at low angles, with peaks at 2.91°, 5.82°, 8.64°, and 11.64°corresponding to the (110), (220), (101), and (330) crystal planes, respectively, indicating an ordered periodic structure. Similarly, the PXRD pattern of Vio-MCOF also displayed characteristic diffraction peaks at 2.96°, 5.92°, 8.79°, and 11.84°, assignable to the (110), (220), (101), and (330) planes (**Figure 1C**), confirming the successful formation of the crystalline MCOF architecture.

**Figure 1:**
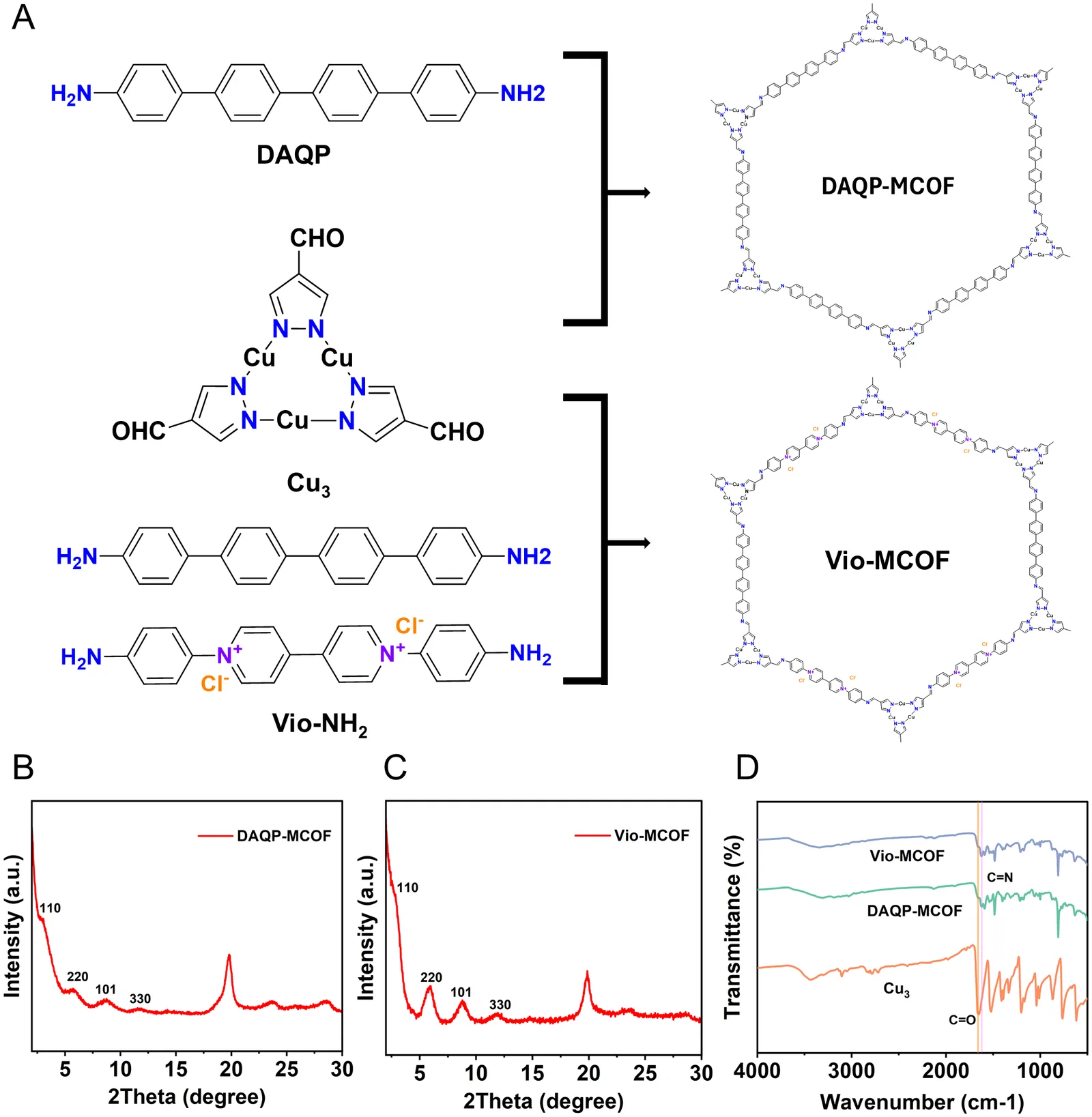
Synthesis and structural characterization of MCOFs. **(A)** Schematic illustration of the solvothermal synthesis of DAQP-MCOF and Vio-MCOF. **(B)** PXRD pattern of DAQP-MCOF. **(C)** PXRD pattern of Vio-MCOF. **(D)** FTIR spectra of Cu_3_, DAQP-MCOF, and Vio-MCOF.

The chemical composition and bonding environment of the materials were further investigated by Fourier transform infrared (FTIR) spectroscopy (**Figure 1D**). The free Cu_3_ precursor exhibited an aldehyde C=O stretching vibration peak at 1662 cm^-1^, whereas this characteristic peak was markedly diminished in DAQP-MCOF and Vio-MCOF. In contrast, new peaks corresponding to the Schiff-base C=N stretching vibration appeared at 1620 cm^-1^ for both DAQP-MCOF and Vio-MCOF, confirming the successful integration of DAQP and Vio-NH_2_ units into the framework structure and indicating the formation of the MCOF networks.

X-ray photoelectron spectroscopy (XPS) analysis confirmed that DAQP-MCOF was composed of C, N, O, and Cu elements (**Figure 2A**), while Vio-MCOF consisted of C, N, O, Cu, and Cl (**Figure 2B**). To further probe the electronic states of the metal centers, high-resolution Cu 2p_3/2_ spectra were acquired for both DAQP-MCOF and Vio-MCOF. As shown in **Figure 2C**, the Cu 2p_3/2_ spectrum of DAQP-MCOF exhibited two major peaks, with Cu present as a mixture of +1 and +2 oxidation states, accompanied by satellite peaks characteristic of open-shell copper centers. In comparison, the Cu 2p_3/2_ spectrum of Vio-MCOF displayed similar features (**Figure 2D**), revealing that the local coordination environment of Cu sites was well-preserved after framework assembly.

**Figure 2:**
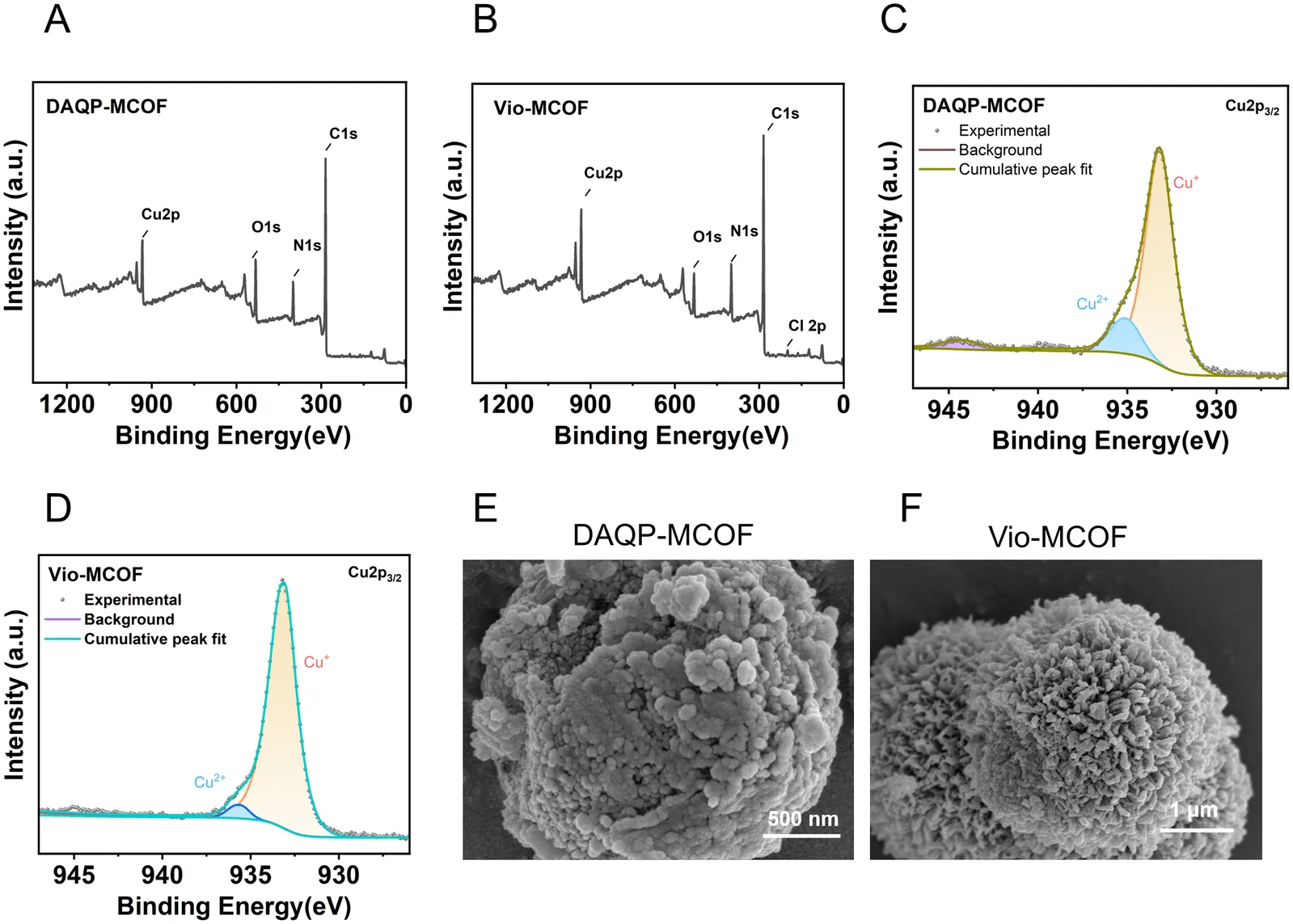
XPS and SEM characterization of DAQP-MCOF and Vio-MCOF. **(A)** XPS survey spectrum of DAQP-MCOF. **(B)** XPS survey spectrum of Vio-MCOF. **(C)** High resolution Cu 2p_3/2_ XPS spectrum of DAQP-MCOF. **(D)** High resolution Cu 2p_3/2_ XPS spectrum of Vio-MCOF. **(E)** SEM image of DAQP-MCOF. **(F)** SEM image of Vio-MCOF.

The surface morphology of the as-synthesized MCOFs was examined by scanning electron microscopy (SEM). As presented in **Figures 2E, F**, both DAQP-MCOF and Vio-MCOF displayed spherical-like agglomerates assembled from primary nanosheet building blocks, with rough surfaces and prominently protruding sheet edges observable. Taken together, these characterizations confirmed the successful construction of two MCOF materials with well-defined crystalline structures and chemical compositions.

### 3.2 Photocatalytic mechanism

UV-vis absorption spectroscopic analysis revealed that both DAQP-MCOF and Vio-MCOF exhibited broad absorption features in the range of 300–1500 nm (**Figure 3A**), indicating excellent light-harvesting capability and potential for solar-driven photocatalytic applications. To evaluate their photodynamic therapy (PDT) performance, the optical band gaps (Eg) were calculated from Tauc plots, yielding values of 2.46 and 1.72 eV for DAQP-MCOF and Vio-MCOF, respectively (**Figure 3B**). Mott-Schottky measurements performed at frequencies of 1500–3000 Hz for DAQP-MCOF and Vio-MCOF displayed positive slopes over the tested potential range (**Figures 3C, D**), which are characteristic of n-type semiconductors. Based on the band position calculations, the conduction band (CB) positions of DAQP-MCOF and Vio-MCOF were determined to be −0.773 and −0.543 eV (vs. NHE), while their valence band (VB) positions were 1.687 and 1.177 eV (vs. NHE), respectively. The band structure diagram (**Figure 3E**) suggests the potential for oxygen reduction to generate O_2_^⁻^, which may subsequently evolve into H_2_O_2_. Transient photocurrent response tests (**Figure 3F**) revealed that Vio-MCOF exhibited a stronger photocurrent response than DAQP-MCOF, indicating that the viologen-functionalized MCOF framework facilitates efficient separation and transport of photogenerated charge carriers.

**Figure 3:**
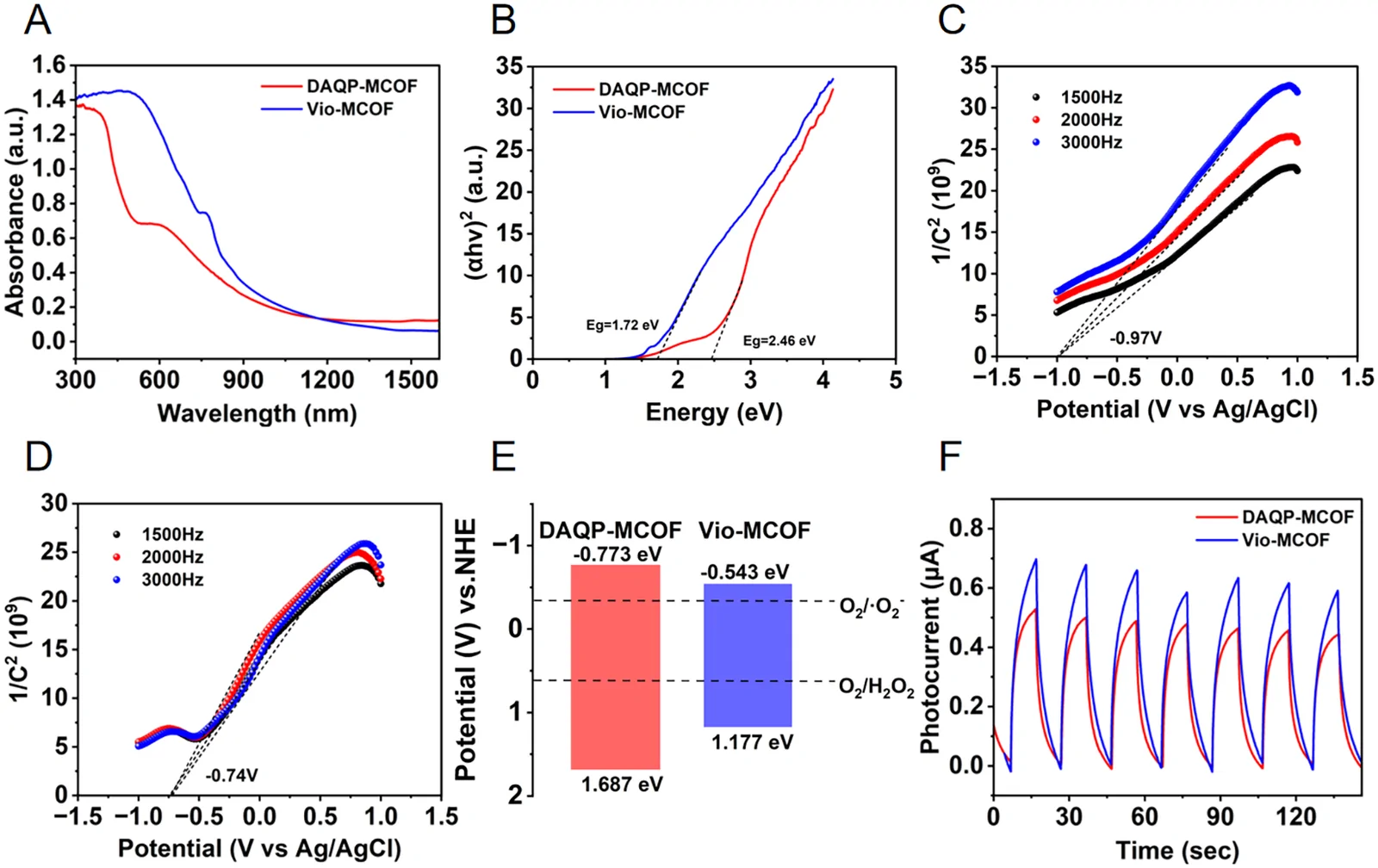
Photophysical and photoelectrochemical characterizations of MCOFs. **(A)** UV-vis-NIR absorption spectra of DAQP-MCOF and Vio-MCOF. **(B)** Tauc plots for band gap estimation. **(C)** Mott-Schottky plots of DAQP-MCOF. **(D)** Mott-Schottky plots of Vio-MCOF. **(E)** Schematic diagram of the band structure and reactive oxygen species (ROS) generation potentials. **(F)** Transient photocurrent response curves of DAQP-MCOF and Vio-MCOF.

### 3.3 *In vitro* antibacterial activity

Motivated by the favorable photoelectronic properties of the as-prepared MCOFs, we further evaluated their antibacterial performance against *S. aureus and E. coli*. As shown in **Figures 4A, B**, under dark conditions, both DAQP-MCOF and Vio-MCOF exhibited concentration-dependent intrinsic antibacterial activity against the two bacterial strains; however, their overall antibacterial effects in the absence of light were limited. Notably, Vio-MCOF showed enhanced antibacterial activity against *S. aureus*, which may be partly attributed to its more positive surface potential (Zeta potential, **Figure 4E**). The positively charged MCOFs can facilitate stronger electrostatic interactions with negatively charged bacterial membranes. Upon light irradiation, both MCOFs exhibited markedly improved antibacterial effects against *S. aureus*. At a concentration of 100 µg mL^-1^, DAQP-MCOF achieved an antibacterial rate exceeding 84.2%, while Vio-MCOF achieved a rate exceeding 96.6%, demonstrating a higher level of antibacterial efficacy (**Figures 4C, D**).

**Figure 4:**
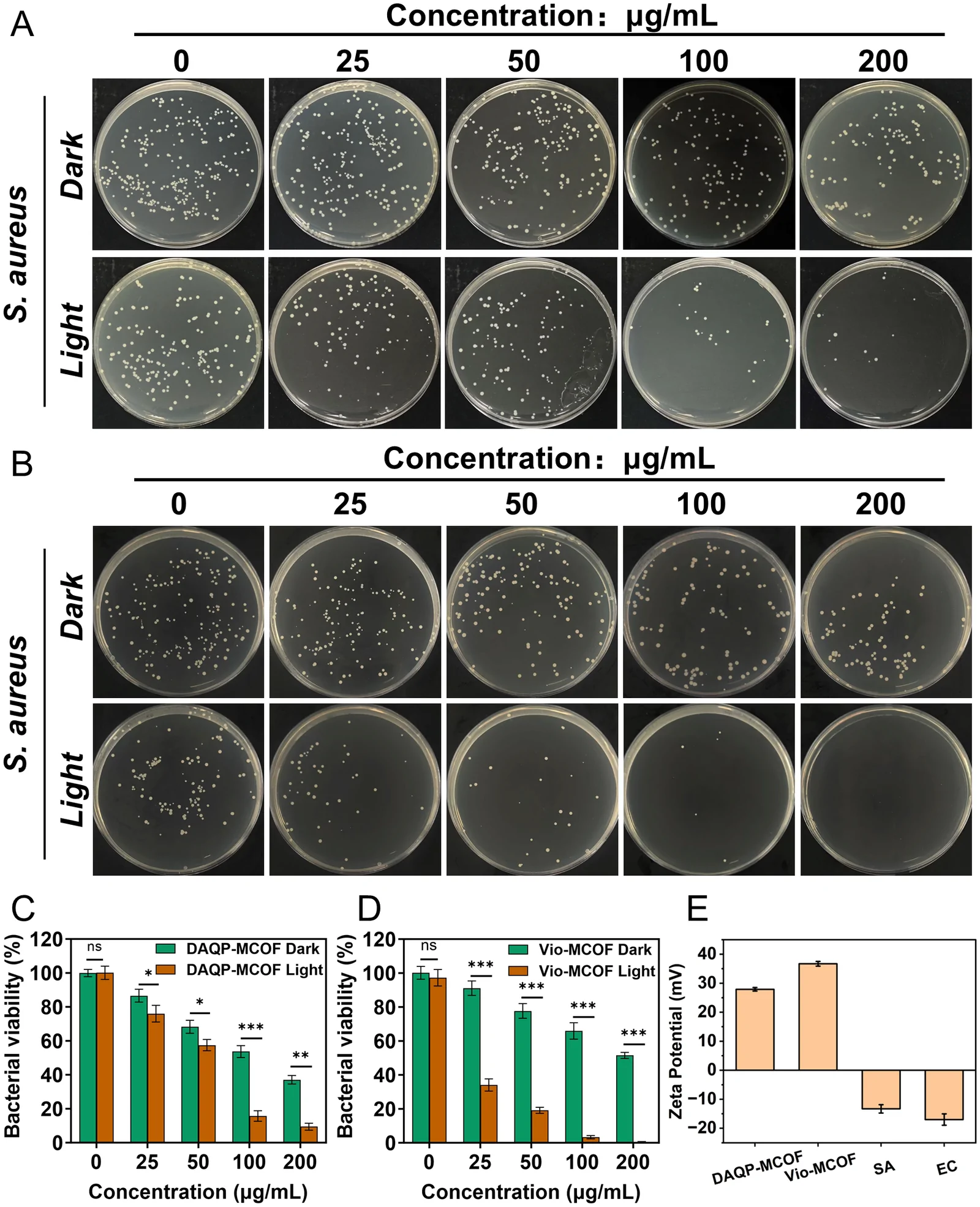
*In vitro* antibacterial activity and surface charge properties of DAQP-MCOF and Vio-MCOF. **(A)** Representative colony photographs of *S. aureus* after incubation with different concentrations of DAQP-MCOF (0-200 μg mL^-1^) under dark and light irradiation. **(B)** Representative colony photographs of *S. aureus* after incubation with different concentrations of Vio-MCOF (0-200 μg mL^-1^) under dark and light irradiation. **(C)** Bacterial viability of *S. aureus* after DAQP-MCOF treatment under dark and light conditions. **(D)** Bacterial viability of *S. aureus* after Vio-MCOF treatment under dark and light conditions. **(E)** Zeta potential measurements of DAQP-MCOF, Vio-MCOF, *S. aureus* and *E. coli*. Data are presented as mean ± SD, n = 3. *p < 0.05, **p < 0.01, ***p < 0.001; ns, no significant difference.

*E. coli* exhibited a similar concentration- and light-dependent antibacterial trend (**Figure 5**). Plate colony-counting results showed that, under light irradiation, the antibacterial activity of DAQP-MCOF against *E. coli* gradually increased with increasing material concentration. At a concentration of 200 μg mL^-1^, the antibacterial rate reached 90.1% (**Figures 5A, B**). In comparison, Vio-MCOF demonstrated superior antibacterial activity against *E. coli*. Under the same irradiation conditions, 200 μg mL^-1^ Vio-MCOF nearly completely eradicated *E. coli*, achieving an antibacterial rate as high as 99.9% (**Figures 5C, D**).

**Figure 5:**
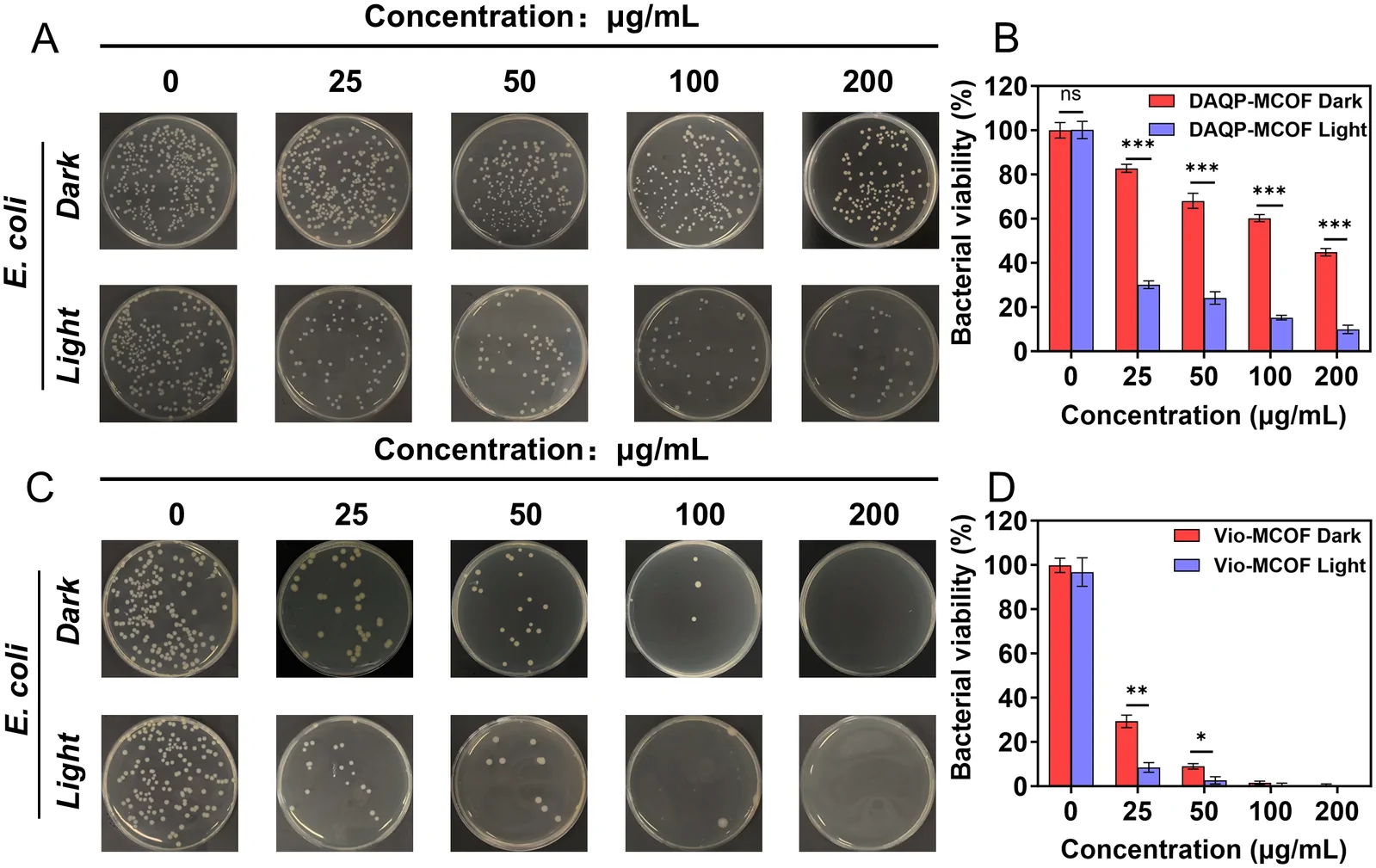
*In vitro* antibacterial activity of DAQP-MCOF and Vio-MCOF against *Escherichia coli*. **(A)** Representative photographs of *E. coli* colonies on agar plates treated with different concentrations of DAQP-MCOF (0-200 μg mL^-1^) under dark and light irradiation. **(B)** Bacterial viability of *E. coli* after DAQP-MCOF treatment under dark and light conditions. **(C)** Representative photographs of *E. coli* colonies on agar plates incubated with different concentrations of Vio-MCOF (0-200 μg mL^-1^) under dark and light irradiation. **(D)** Bacterial viability of *E. coli* after Vio-MCOF treatment under dark and light conditions. Data are presented as mean ± SD, n = 3. *p < 0.05, **p < 0.01, ***p < 0.001; ns, no significant difference.

### 3.4 Synthesis and antibacterial activity of MCOF-based spray hydrogel

To translate the excellent photocatalytic antibacterial performance of the synthesized MCOFs into a practically applicable formulation, we further incorporated them into a sprayable gel system. The gelation process was first visually inspected. As shown in **Figure 6A**, the precursor solutions of different gel formulations remained as free flowing liquids before gelation. Upon the addition of calcium chloride solution, rapid crosslinking occurred within the precursor solutions, inducing a sol-gel transition and resulting in structurally stable, self-supporting gels with good shape retention, indicating the successful construction of a sprayable and *in situ* gelforming platform suitable for topical application.

**Figure 6:**
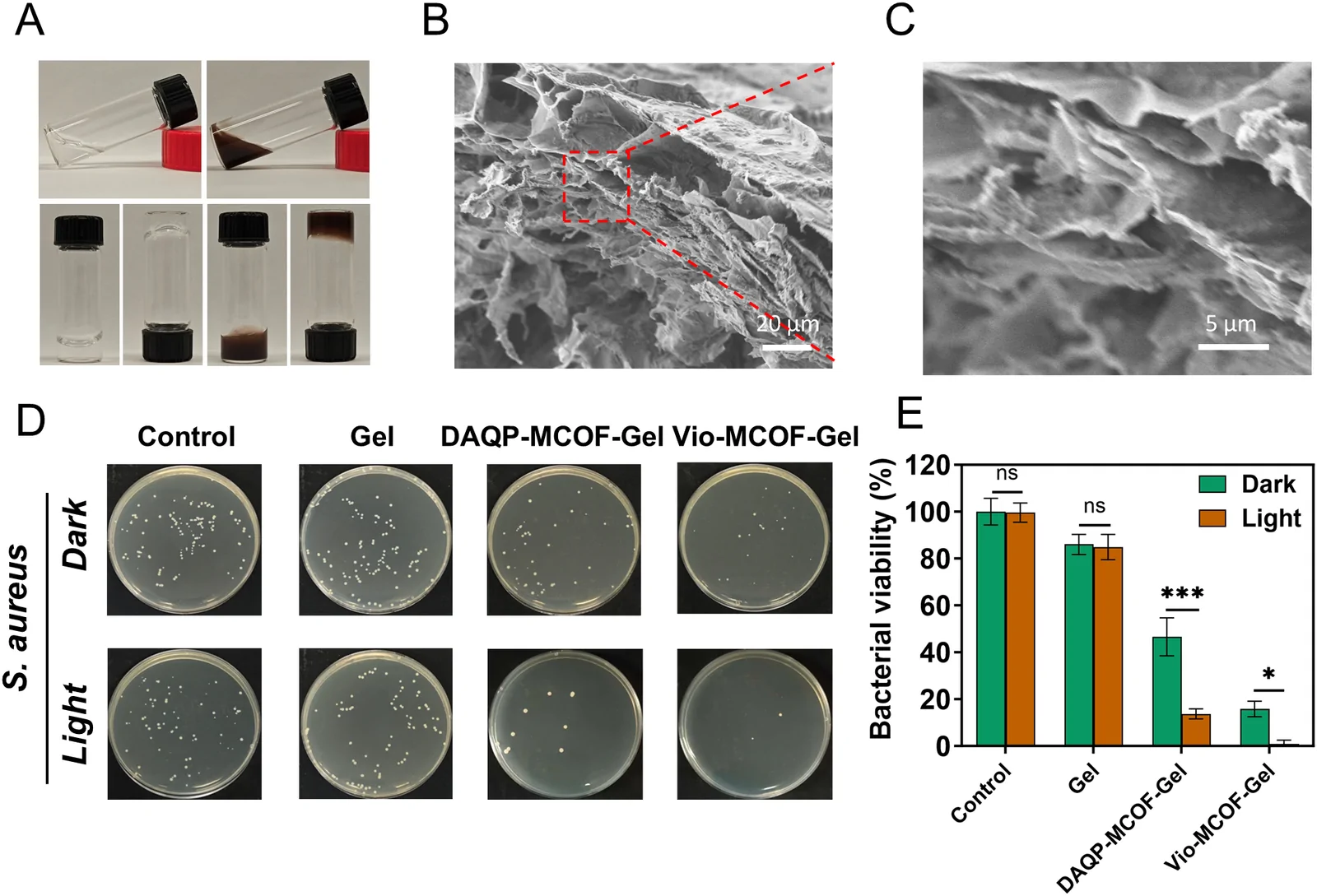
Characterization and antibacterial activity evaluation of MCOF-based sprayable hydrogel. **(A)** Photographs of different hydrogels before and after gelation. **(B)** SEM image of the Vio-MCOF gel (scale bar: 20 μm). **(C)** High-magnification SEM image of the Vio-MCOF gel (scale bar: 5 μm). **(D)** Representative photographs of *S. aureus* colonies formed on LB agar plates under different treatments in dark and light conditions. **(E)** Corresponding bacterial viability of *S. aureus* for each group. Data are presented as mean ± SD, n = 3. *p < 0.05, ***p < 0.001; ns, no significant difference.

The microstructure of the representative Vio-MCOF hydrogel was characterized by SEM. As presented in **Figure 6B**, the freezedried gel exhibited a highly porous, three-dimensional interconnected network with a uniform pore size distribution. At higher magnification (**Figure 6C**), MCOF nanoparticles were clearly observed to be uniformly embedded within the polymer wall matrix, without obvious aggregation or phase separation. The well-dispersed incorporation of MCOFs in the porous hydrogel scaffold not only preserved their photocatalytic activity by ensuring adequate light transmission and mass transfer, but also facilitated bacterial capture and contact killing upon local application.

Based on the superior structural properties of the composite hydrogels, we further evaluated the *in vitro* photodynamic antibacterial activity of DAQP-MCOF-Gel and Vio-MCOF-Gel against *S. aureus*. The bacterial colony growth on agar plates in each group is shown in **Figure 6D**. The blank control group exhibited a large number of bacterial colonies with good growth. In contrast, the Gel group showed no obvious reduction in colony density, regardless of whether light irradiation was applied, confirming that the blank hydrogel matrix itself possessed little intrinsic antibacterial activity.

Under dark conditions, the MCOF-loaded composite hydrogels already exhibited a certain degree of basal antibacterial activity, as evidenced by the reduced number of colonies compared with the pure hydrogel group. Following light irradiation, bacterial colonies in both the DAQP-MCOF-Gel and Vio-MCOF-Gel groups decreased markedly (**Figure 6D**). Quantitative results (**Figure 6E**) were consistent with the agar plate observations. No significant difference in bacterial survival was observed between the control and pure hydrogel groups. After light activation, the antibacterial activity of composite hydrogels was significantly enhanced. The antibacterial rates of DAQP-MCOF-Gel and Vio-MCOF-Gel against *S. aureus* reached 86.3% and 99.1%, respectively. Vio-MCOF-Gel exhibited significantly better photodynamic antibacterial efficacy than DAQP-MCOF-Gel upon light irradiation.

Collectively, these results demonstrate that the MCOF-based composite hydrogels successfully retained the excellent photodynamic antibacterial activity of the MCOF powder, while light irradiation further amplified their bactericidal efficacy. Upon light activation, the composite hydrogels efficiently inactivated *S. aureus*, indicating their considerable potential as photoresponsive antibacterial wound dressings for the treatment of infected wounds.

### 3.5 *In vitro* biosafety of Vio-MCOF hydrogel

Biosafety is a fundamental prerequisite for the *in vivo* application of biomaterials. In this study, the *in vitro* biocompatibility of the Vio-MCOF-Gel composite hydrogel was evaluated using an L929 fibroblast CCK-8 cell proliferation assay and a hemolysis assay. The Vio-MCOF powder inhibited L929 cell proliferation at relatively high concentrations (50 and 100 μg mL^-1^). However, the hydrogel matrix effectively attenuated this cytotoxicity, and the Vio-MCOF-loaded composite hydrogel did not cause a significant reduction in cell viability (**Figures 7A, B**). The hemolysis assay showed that the hemolysis rates of all material groups were below the safety threshold of 5%, indicating that these materials did not induce significant erythrocyte lysis and exhibited good hemocompatibility (**Figures 7C, D**). Collectively, these results demonstrate that the Vio-MCOF-Gel composite hydrogel possesses excellent *in vitro* biosafety and shows promise for the treatment of tissue injuries associated with bacterial infections.

**Figure 7:**
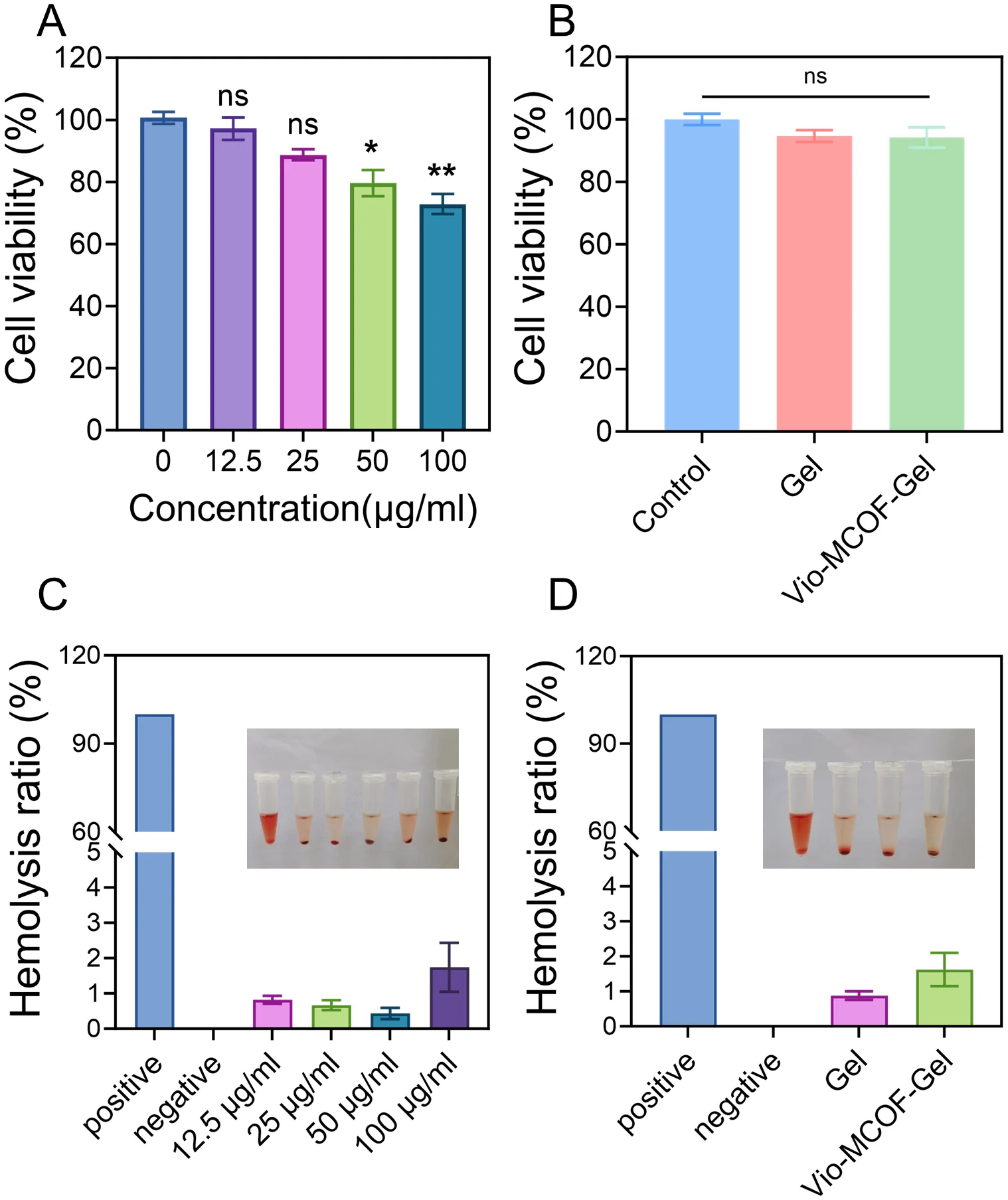
*In vitro* biocompatibility evaluation of the Vio-MCOF powder and hydrogel. **(A)** Cell viability of L929 fibroblasts after incubation with different concentrations of Vio-MCOF powder. **(B)** Cytocompatibility of L929 cells treated with blank hydrogel and Vio-MCOF hydrogel. **(C)** Hemolysis ratios of red blood cells incubated with different concentrations of Vio-MCOF powder. **(D)** Hemolysis ratios of red blood cells incubated with blank hydrogel and Vio-MCOF hydrogel. Data are presented as mean ± SD, n = 3. *p < 0.05, **p < 0.01; ns, no significant difference.

### 3.6 *In vivo* therapeutic performance of Vio-MCOF hydrogel for bacteria-infected skin wounds

To further evaluate the role of the Vio-MCOF hydrogel in skin wound healing, a full thickness skin defect model infected with *S. aureus* was established on the dorsal skin of mice, and the overall treatment protocol is illustrated in **Figure 8A**. Wound photographs were taken and the wound closure rates were calculated on days 0, 3, 6, 8, and 10. The results showed that the wound closure rate was significantly accelerated in the Light-Gel group, whereas the infected wounds in the other groups healed slowly (**Figures 8B, E**). To further evaluate the *in vivo* antibacterial performance of the material, wound tissues were collected on day 3 post treatment, homogenized, plated for culture, and subjected to colony counting. Compared with the Control group, the number of bacterial colonies on the plates was markedly reduced in both the Dark-Gel group and the Light-Gel group, with the Light-Gel group exhibiting the lowest bacterial survival rate (**Figures 8C, D**). These findings confirm that the hydrogel possesses robust antibacterial activity, and that light irradiation can further enhance its efficacy in clearing *S. aureus* within the wound.

**Figure 8:**
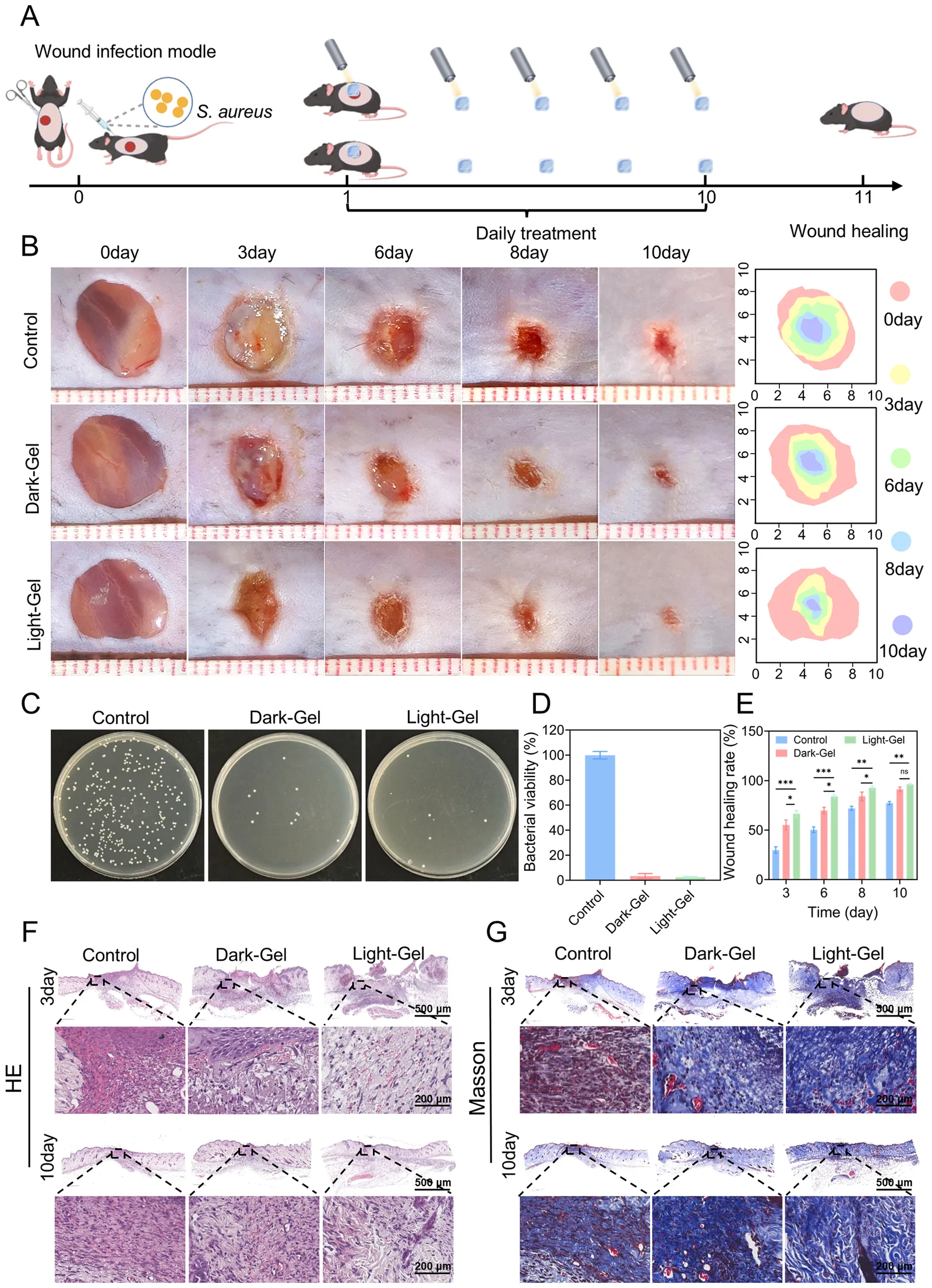
*In vivo* evaluation of the therapeutic performance of different treatment groups for infected wounds. **(A)** Schematic diagram illustrating the animal experimental procedure for infected wound modeling and treatment. **(B)** Gross wound photographs and corresponding wound area overlay images of different groups obtained on days 0, 3, 6, 8, and 10. **(C)** Representative LB agar plates showing the *in vivo* antibacterial effects of different groups on day 3 post-treatment. **(D)** Bacterial viability of *S. aureus* in wound tissues on day 3. **(E)** Statistical analysis of wound healing rates for each treatment group. **(F)** H&E stained wound tissue sections from different groups on days 3 and 10. **(G)** Masson’s trichrome stained wound tissue sections from different groups on days 3 and 10. Scale bars: 500 μm (low-magnification), 200 μm (high-magnification). Data are presented as mean ± SD, n = 5 mice per group. *p < 0.05, **p < 0.01, ***p < 0.001; ns, no significant difference.

Histological analyses were performed on days 3 and 10 to further assess the quality of regenerated skin. H&E staining revealed that on day 3, the control group exhibited massive inflammatory cell infiltration in the wound, whereas the Light-Gel group displayed fewer infiltrating inflammatory cells. On day 10, although inflammatory cells were reduced in the control group, they remained abundant, and the dermal and epidermal structures were barely formed; in contrast, the Light-Gel group exhibited markedly reduced inflammatory cell infiltration in the wound (**Figure 8F**). Masson’s trichrome staining demonstrated that the Light-Gel group had orderly and densely deposited collagen fibers (**Figure 8G**).

Furthermore, immunofluorescence staining was used to detect the expression of the pro-inflammatory cytokines TNF-α and IL-6 in wound tissues. The Light-Gel group exhibited lower expression levels of TNF-α and IL-6 compared with the other two groups, suggesting mitigated inflammatory infiltration (**Figures 9A, B, D, E**). Meanwhile, immunohistochemical staining of the angiogenesis marker CD31 was performed on wound tissues. As shown in **Figures 9C, F**, CD31 expression was markedly up-regulated in the Light-Gel group. Collectively, these results indicate that the light-activated Vio-MCOF hydrogel can substantially accelerate infected wound repair.

**Figure 9:**
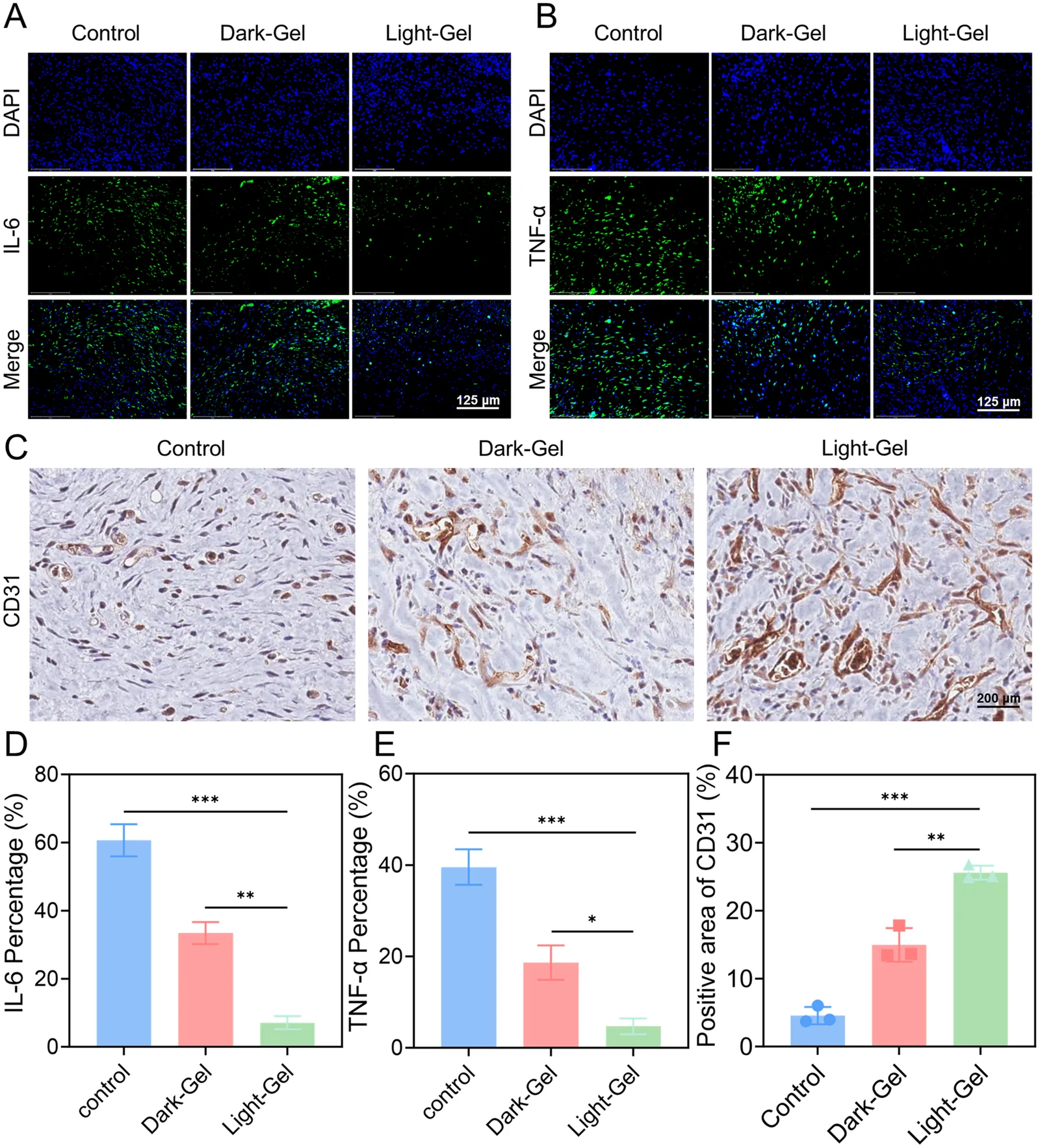
*In vivo* evaluation of inflammatory response and angiogenesis in wound tissues. **(A)** Representative immunofluorescence staining of the pro-inflammatory cytokine IL-6 in wound tissues from different groups on day 3. **(B)** Representative immunofluorescence staining of TNF-α in wound tissues from different groups on day 3. **(C)** Representative immunohistochemical staining of CD31 in wound tissues from different groups on day 3. **(D)** Quantitative analysis of the relative positive area of IL-6. **(E)** Quantitative analysis of the relative positive area of TNF-α. **(F)** Quantitative analysis of the relative positive area of CD31. Scale bars: 125 μm **(A, B)**, 200 μm **(C)**. Data are presented as mean ± SD (n = 3). *p < 0.05, **p < 0.01, and ***p < 0.001.

## 4 Discussion

Infected wounds are usually associated with bacterial overgrowth, sustained inflammation, poor granulation tissue formation, and delayed re-epithelialization and collagen deposition. As a result, dressings that combine antibacterial, anti-inflammatory, and pro-healing functions are needed to improve the repair of infected wounds (Almadani et al., 2021; Liu et al., 2023a; Zhou et al., 2025c). Instead of physically doping or surface-loading photosensitizers, viologen units were incorporated directly into the MCOF framework during synthesis to obtain Vio-MCOF. MCOFs combine the catalytic advantages of metal active sites derived from MOFs with the robust covalent skeletons of COFs, and thus maintain abundant catalytic centers and show improved structural stability under physiological conditions (Zhang et al., 2025). Viologen units show reversible redox-driven electron-transfer capacity and intrinsic positive charge. They act as electron-trapping sites, suppress photogenerated electron-hole recombination, and increase ROS production (He et al., 2024; Qi et al., 2024). Cationic viologen groups bind to negatively charged bacterial membranes, improving material attachment on pathogen surfaces. Compared with free viologen molecules or surface-loaded photosensitizers, this covalent embedding strategy reduces leaching and improves stability while retaining photocatalytic and bacterial-binding functions (Zhou et al., 2025b; Mi et al., 2021).

XRD, FTIR, XPS, and SEM confirmed that viologen units were covalently incorporated into the MCOF framework via aldehyde-amine condensation. Compared with DAQP-MCOF, Vio-MCOF preserved the original crystal phase and copper-cluster active sites and maintained a mixed Cu^+^/Cu^2+^ valence state, which provided abundant catalytic metal centers for efficient photogenerated charge transfer (Liu et al., 2023b). Both samples self-assembled into spherical aggregates stacked from nanosheets. This hierarchical structure provides a large specific surface area, facilitating light capture, charge transport, and contact with bacterial cells. Zeta potential measurements showed that Vio-MCOF exhibited a significantly higher positive surface charge than DAQP-MCOF. This increased positive surface charge originates from cationic viologen moieties and promotes electrostatic binding to bacterial surfaces.

We further explored whether viologen incorporation could boost the photodynamic performance of Vio-MCOF via optical and electrochemical characterizations. Relative to DAQP-MCOF, Vio-MCOF displayed a narrowed band gap, broader absorption under simulated solar light, and better light utilization. Many Cu-containing COFs/MOFs suffer from fast carrier recombination and weak response to simulated solar light. Most of them require ultraviolet excitation for activation, which limits their *in vivo* applications. Mott-Schottky tests indicated that both materials behaved as n-type semiconductors, and their band positions satisfied the thermodynamic requirement for O_2_ reduction to generate superoxide radicals. Transient photocurrent results showed that Vio-MCOF produced a much stronger photocurrent response than DAQP-MCOF. This finding suggests that anchored viologen traps photogenerated electrons, suppresses electron-hole recombination and facilitates electron transfer pathways with copper cluster sites. Together, viologen-mediated electron trapping and copper-cluster catalysis are proposed to improve ROS generation. Vio-MCOF also showed weaker dependence on ultraviolet excitation, making it more suitable for wound environments.

*In vitro* antibacterial assays demonstrated that the antibacterial activity of Vio-MCOF was mainly light-dependent. In the dark, both DAQP-MCOF and Vio-MCOF showed mild concentration-dependent bacteriostatic effects against *S. aureus*, and this behavior was consistent with previous reports (He et al., 2024; Dong et al., 2025; Zhao et al., 2025). Upon simulated solar light irradiation, Vio-MCOF exhibited significantly enhanced bactericidal rate against *S. aureus*. At 200 μg mL^-1^, Vio-MCOF achieved over 99% bactericidal rate against *S. aureus* after 30 min of irradiation, which was higher than that of DAQP-MCOF. These results suggest that, under the tested light conditions, light-triggered ROS generation likely contributed to the enhanced antibacterial activity. Zeta potential analysis further showed that Vio-MCOF possessed a higher positive surface charge than DAQP-MCOF, while the cell membranes of *S. aureus* were negatively charged. Therefore, Vio-MCOF can achieve enhanced adhesion to bacterial surfaces via electrostatic attraction. This effect shortens the distance between ROS and bacterial targets and improves photodynamic killing efficacy. In addition, the residual bacteriostatic activity observed in the dark may arise partly from potential copper-ion release and the cationic surface charge of Vio-MCOF. Overall, Vio-MCOF showed limited antibacterial activity in the dark but strong light-triggered aPDT. This antibiotic-free approach may help reduce the risk of bacterial resistance.

A sprayable ionically cross-linked hydrogel was fabricated by incorporating Vio-MCOF into a sodium alginate matrix. The hydrogel formed *in situ* when Ca^2+^ crosslinked alginate carboxylates. This allows it to be sprayed onto irregular wounds and fill areas that preformed dressings cannot easily cover. Compared with most preformed solid dressings, sprayable systems showed better conformability to irregular wound beds and were more suitable for the initial management of infected wounds (Zhou et al., 2025a; Kibungu et al., 2021; Liu et al., 2023d). SEM images of the lyophilized hydrogel showed an interconnected three-dimensional porous network, with Vio-MCOF particles homogeneously dispersed without obvious aggregation. The porous structure facilitates light penetration, exudate absorption, and gas exchange, thereby creating a favorable microenvironment for aPDT. Meanwhile, the sodium alginate hydrogel acts as a flexible carrier to immobilize Vio-MCOF and prevent material loss. This improved the stability of the material inside the wound.

We further evaluated the *in vivo* healing potential of this composite hydrogel using a full-thickness skin wound model infected with S. aureus. Compared with the control groups, the Light-Gel group achieved faster wound closure and a lower bacterial burden. This result suggests that the hydrogel can reduce *S. aureus* colonization *in vivo* and suppress bacteria-provoked inflammatory responses. Immunofluorescence and histological analyses demonstrated lower expression of pro-inflammatory factors TNF-α and IL-6 in the Light-Gel group. In addition, this group displayed a higher density of CD31-positive microvessels and more organized and denser collagen deposition. These findings reveal that the Vio-MCOF hydrogel under simulated solar light reduced bacterial burden and inflammation while promoting angiogenesis and tissue repair. Notably, the Dark-Gel group also showed improved healing relative to the blank control group. This improvement likely arises mainly from the physical barrier and moisture-retention properties of the sodium alginate hydrogel matrix. The weak intrinsic bacteriostatic effect of embedded Vio-MCOF under dark conditions may provide auxiliary benefits, although this effect was marginal in our *in vitro* antibacterial assays of the hydrogel. The additional improvement observed under irradiation suggests that light-activated antibacterial activity likely contributed to the improved *in vivo* antibacterial efficacy. Compared with many small-molecule photosensitizers or surface-loaded photocatalysts (Zhao et al., 2022; Dong et al., 2025; Zhou et al., 2025b), the present system integrates electron-transfer modulation and bacterial capture through covalently embedded viologen units. Under simulated solar light, it showed effective antibacterial and tissue-repair performance. Notably, the Vio-MCOF-loaded hydrogel exhibited good biocompatibility toward L929 fibroblasts, whereas free Vio-MCOF induced concentration-dependent cytotoxicity at high doses. The alginate matrix likely restricts Vio-MCOF release, keeping the local concentration below the cytotoxic threshold. By immobilizing Vio-MCOF within the hydrogel, this strategy mitigates the cytotoxicity associated with free powder and supports its potential use in *in vivo* phototherapy.

This study has several limitations. First, our therapeutic evaluation was carried out only in an acute *S. aureus*-infected full-thickness wound model. We did not test the material in more challenging chronic settings, such as diabetic or ischemic wounds. Further evaluation in complex chronic wound models is needed to validate its reparative efficacy and translational potential. Second, direct ROS detection was not performed in this work, and the effects of light-irradiation conditions and material dosage on long-term antibacterial and antibiofilm performance still need to be examined in detail. Third, the *in vivo* degradation behavior, metabolic products, and long-term biosafety of Vio-MCOF also require further investigation. In future studies, we will optimize hydrogel formulations and light-treatment parameters to extend this system to the management of chronic infected wounds.

## 5 Conclusions

In the present study, viologen units were embedded *in situ* into the MCOF framework to construct Vio-MCOF with strong bacterial adhesion capability and efficient photodynamic antibacterial performance. A sprayable wound hydrogel dressing was developed by integrating Vio-MCOF into an ionically cross-linked sodium alginate network. The rigid covalent skeleton of the MCOF stably immobilizes copper-cluster active sites, thereby improving photocatalytic stability and charge-separation efficiency. Due to the intrinsic positive charge of the viologen moieties, Vio-MCOF preferentially adheres to bacterial surfaces through electrostatic interactions. Such adhesion reduces the distance between ROS-generating sites and bacterial targets. In addition, the covalently embedded viologen units act as efficient electron mediators and synergize with copper clusters to significantly enhance ROS generation, thereby producing efficient *in situ* bactericidal activity at infected wound sites. Our *in vitro* and *in vivo* experiments confirm that this composite hydrogel suppresses bacterial proliferation, modulate the wound inflammatory microenvironment, promotes collagen deposition and angiogenesis, and ultimately accelerate the healing of infected skin wounds. Therefore, the Vio-MCOF-based sprayable hydrogel is a promising dressing for managing bacterially infected skin wounds.

## Data Availability

The original contributions presented in the study are included in the article, further inquiries can be directed to the corresponding author.
